# Mpox Virus: Control of In-Hospital Occupational Transmission Experience from a Tertiary Level Hospital in Milan, Italy

**DOI:** 10.3390/life13081705

**Published:** 2023-08-08

**Authors:** Angelo Roberto Raccagni, Nicola Gianotti, Matteo Moro, Davide Mileto, Victoria Gordo Perez, Antonella Castagna, Silvia Nozza

**Affiliations:** 1 Infectious Diseases Unit, Vita-Salute San Raffaele University, 20132 Milan, Italy; castagna.antonella1@hsr.it (A.C.); nozza.silvia@hsr.it (S.N.); 2 Infectious Diseases Unit, San Raffaele Scientific Institute, 20132 Milan, Italy; gianotti.nicola@hsr.it (N.G.); gordo.victoria@hsr.it (V.G.P.); 3 Infection Control, Chief Medical Office, San Raffaele Scientific Institute, 20132 Milan, Italy; moro.matteo@hsr.it; 4 Laboratory of Clinical Microbiology, Virology and Bioemergencies, L. Sacco University Hospital, 20122 Milan, Italy; mileto.davide@asst-fbf-sacco.it

**Keywords:** mpox, transmission, occupational, virus, prevention, monkeypox

## Abstract

Mpox has caused a global outbreak since May 2022, particularly affecting people belonging to key populations, but cases among healthcare providers have been reported. The aim of this work is to present the experience of the Infectious Diseases Unit of San Raffaele Scientific Institute, Milan, Italy with respect to infection control and prevention of mpox occupational transmission. Between May–November 2022, 140 individuals were diagnosed with mpox and six required hospitalization. Overall, 12 medical doctors and 22 nurses provided care to people with mpox. A hospital policy aimed at controlling viral transmission was implemented in May 2022. Protective equipment was used for all healthcare providers. One accidental puncture occurred with a scalpel contaminated with blood from a mpox viremic individual (mpox plasma cycle threshold = 36); no mpox related symptoms were observed and mpox testing ruled out transmission. Six months following exposure, neutralizing antibodies were not detectable, ruling out contagion. Overall, we observed no mpox transmission among healthcare workers, despite the number of visits and procedures performed, including bodily-fluids sampling, and even following puncture with contaminated blood. Hospital preparedness for the management of new infectious disease outbreaks, with rapid implementation of policies aimed at controlling infection, is paramount to avoid occupational transmission.

## 1. Introduction

Mpox (formerly Monkeypox virus), an Orthopoxvirus, has caused a global outbreak since May 2022, which started spreading in Europe and North America, leading to the declaration by the World Health Organization (WHO) of mpox as a public health emergency of international concern (PHEIC) [1,2,3,4]. During the current outbreak, human-to-human transmission was the predominant way of viral transmission outside the African endemic region [1,2,3,4]. Infections have been reported among people belonging to key populations [1,2,3,4]. Mpox can be transmitted from infected animals to humans by bite, scratch, exposure to animal blood or bodily fluids, prolonged close contact and by eating undercooked animal meat [5,6,7]. Human-to-human transmission routes are heterogeneous, since one can be infected through inhalation of respiratory droplets after prolonged face-to-face contact, contact with bodily fluids or infectious lesions, contaminated fomites and vertical transmission [1,7,8]. However, in the current mpox outbreak, the virus was proven to transmit between humans following close contact with infected individuals and, more often, due to sexual exposure [1,2,3,4].

The likelihood of pure sexual transmission is still uncertain as of today, despite the fact that most infected persons during the 2022 epidemic reported at-risk sexual behavior before the onset of signs and symptoms and that lesions were strongly associated with the site of sexual contact (anogenital area) [1,2,3,4,9,10]. In this particular case, the consensus is that sexual activity, due to the generation of microscopic abrasions or sores in mucous membranes, may facilitate viral transmission. Replication-competent mpox virus has been detected in semen samples, regardless of the presence of genital lesions [11,12,13]. Other than semen, replication-competent mpox was isolated from skin and oropharyngeal swabs [14,15]. Mpox DNA, independent of the viral capability to replicate, was detected in skin, oropharyngeal mucosa, semen, urine, feces and saliva, with the highest viral loads being consistently found in biological specimens coming from cutaneous lesions [1,2,3,4]. These data support that sexual transmission, including close skin-to-skin contact and bodily fluids, contributed to the outbreak. Moreover, infections among household members of infected individuals were also described [16]. Regarding mpox, cases of infection after a needle-stick injury have been reported in healthcare professionals [17,18]. Moreover, infections following contact without the use of protective equipment were also described [19,20]. Given these premises, some countries, including the US, recommend vaccination with Modified Vaccinia Ankara Bavarian Nordic (MVA-BN) as pre-exposure prophylaxis for healthcare providers caring for individuals diagnosed with mpox [21]. Post-exposure vaccination with MVA-BN is also recommended for individuals, including healthcare workers, with possible exposure to the virus [22,23]. In Italy, the MVA-BN vaccination was implemented in August 2022 and was recommended to people belonging to key populations and laboratory personnel handling infected specimens. The availability of vaccines from late August 2022 in Italy did not allow using the post-exposure vaccination strategy from the very beginning of the epidemic [24]. Occupational transmission of infections is a growing problem, especially for healthcare providers working with infectious diseases or caring for fragile individuals. This might endanger the well-being of workers or result in a loss of workforce, which is paramount to addressing a new emerging pathogen. This was particularly true for the SARS-CoV-2 epidemic, where healthcare providers were at the front of the pandemic and were particularly at risk of being infected [25]. Policymakers, including local infection control teams, coordinated by regional and national institutions, play a pivotal role in rapidly addressing these risks, by delivering and proposing infection control policies and guiding all healthcare providers on how to correctly prevent further pathogen transmission and avoid occupational infection.

The aim of this article is to present the experience of the Infectious Diseases Unit of San Raffaele Scientific Institute, Milan, Italy, regarding infection control and prevention of occupational transmission of mpox virus during the 2022 outbreak. 

## 2. Materials and Methods

Individuals suspected to have the mpox infection received medical care by accessing the walk-in Sexual Health Clinic of the Infectious Diseases Unit of San Raffaele Scientific Institute, Milan, Italy, which is mainly dedicated to individuals who want to receive sexually transmitted infections (STI) tests for periodical screening, the presence of suspected symptoms, HIV pre-exposure prophylaxis (PrEP) users, and people who need to take HIV post-exposure prophylaxis (PEP). At first access, we considered as a suspected diagnosis of mpox every individual who presented symptoms consistent with an infection. We also considered those who met the epidemiological criteria and had a high clinical suspicion for mpox. According to the Case Reporting Recommendations for Health Departments released by the Centers for Disease Control and Prevention (CDC), epidemiological criteria included a “history of close, intimate contact with people with a similar appearing rash or who received a diagnosis of confirmed or probable mpox or close or intimate in-person contact with individuals in a social experiencing mpox activity, including MSM or social event or traveled to a country endemic or with confirmed cases of mpox” [23]. In these cases, we performed a physical examination and researched mpox via oropharyngeal, anal, genital and cutaneous swabs, plasma, serum, urine and semen samples. Individuals with confirmed mpox diagnosis were re-evaluated once per week approximately. Healthcare workers repeated clinical evaluation and collection of specimens for virology analyses, based upon medical judgment. Medical doctors performed physical examinations, including skin and genital area inspection, oropharyngeal examination and collection of specimens for PCR testing (including oropharyngeal, anal, genital and lesions swabs). In order to effectively collect viral material, skin lesions were tested, based on clinical judgment, following the scraping of the lesion with a scalpel. Nurses collected blood in plasma and serum tubes and handled all samples for delivery. Virology analyses were performed at the Laboratory of Clinical Microbiology, Virology and Bioemergencies of *Luigi Sacco* University Hospital, Milan, an Italian reference center for mpox diagnosis. Samples were shipped in dedicated bio-carriers for biologic specimens (category B, UN3373), with triple packaging to avoid viral dissemination in case of accidence during delivery. Nurse personnel conducted the packaging and handling of clinical specimens in a dedicated room. Overall, 14 medical doctors and 7 nurses provided care to individuals diagnosed with mpox in the outpatient setting. Regarding individuals hospitalized, 22 nurses and 8 medical doctors provided care with multiple reassessments every day, specimen and blood collection.

According to the international guidelines (CDC and ECDC), the Infection Control Team published early (24 May 2022) and then updated four times the internal document “Vaiolo delle scimmie (monkeypox): gestione dei casi sospetti o accertati” for management of suspected or diagnosed cases of mpox: healthcare workers providing care for individuals with suspected or confirmed mpox implemented infection control precautions, including standard, droplet and contact ones, with particular focus on hand hygiene [26,27]. The first mpox case diagnosed at our hospital was on the 25th of May 2022. Personal protective equipment (PPE), i.e., gown, gloves and respirator (due to the ongoing COVID-19 pandemic) were used by all personnel when visiting individuals or handling all specimens. Individuals with suspected mpox infection used facemasks during medical visits, if tolerated or not contraindicated. A dedicated waiting room and a dedicated consultation room were used; no administrative clearance was required in order to avoid contact between suspected mpox cases and other people visiting the hospital. Environmental cleaning and disinfection of the room following patients’ discharge in the in-patient setting, as well as accurate reprocessing of all used medical devices, was conducted. Soiled laundry (e.g., bedding) was handled in accordance with standard practices, avoiding contact with lesion material that may be present on the laundry and handled in a manner that does not disperse infectious material. Management of food service items was performed in accordance with routine procedures. Accurate cleaning and disinfection of the involved environmental surfaces at every medical visit or sample collection were performed by dedicated personnel, with virucide agents active against mpox (a chlorine compound in high concentrations i.e., >1000 ppm). Patients were considered infectious and infection control measures were applied until the complete resolution of clinical symptoms, including scabs fall.

According to hospital policy, all healthcare workers were monitored for mpox-consistent symptoms development. Referring in particular to those exposed to mpox without wearing PPE or in case of accidents, it was indicated that they monitor themselves for fever by taking their temperature twice a day and remain alert for any suspect symptoms for 21 days: Only one accident occurred, as later discussed. For cases of suspected mpox or in order to exclude occupational mpox infection among healthcare workers with high-risk viral exposure, a real-time polymerases chain reaction (RT-PCR) (RealStar^®^ Orthopoxvirus PCR Kit 1.0—Altona Diagnostics) targeting the *variola* virus and non-variola Orthopoxvirus species (cowpox, mpox, raccoonpox, camelpox, *vaccinia virus*) was used to detect the presence of non-variola DNA on rectal, oropharyngeal and lesions swabs, urine, plasma and seminal fluids. Cycle thresholds (Ct) for positive samples of the Orthopoxvirus PCR test are presented. A specific RT-PCR targeting mpox DNA (Jiangsu Bioperfectus Technologies Co., Ltd., Taizhou, China) was subsequently used to confirm the presence of mpox on the specimen with the lowest Ct. Rectal, oropharyngeal and lesions swabs were collected with Universal Transport Medium swabs (UTM-RT; COPAN Diagnostics, Brescia, Italy). Urine and seminal fluid specimens were collected using sterile screw cap containers. Following possible mpox occupational transmission, a plaque reduction neutralization test (PRNT) was used to assess the presence of neutralizing anti-mpox antibodies in the serum after 6 months from exposure. Briefly, 50 μL of each serum, starting from a 1:10 dilution followed by serial two-fold series, were transferred in two wells of 96-weel microtiter plates (COSTAR, Corning Incorporated, Corning, NY 14831, USA) and mixed with 50 µL of tissue culture infecting dose 50 (TCID50) of mpox virus (EPI_ISL_13302316). All dilutions were made in DMEM with 1% penicillin and streptomycin. After one-hour incubation at 37 °C and 5%CO_2_, 50 µL of 2 × 10^4^ VeroE6 (VERO-C1008-ATCC^®^-CRL-1586™) cells were added to each well. After 6 days of incubation at 37 °C and 5%CO_2_, wells were stained with 0.1% crystal violet solution (Merck KGaA, 64271 Darmstadt, Germany) plus 5% formaldehyde 40% *m*/*v* (Carlo ErbaSpA, Arese, Italy) for 30 min; microtiter plates were washed in running water. Wells were scored to evaluate the degree of cytopathic effect (CPE) compared to the virus control; blue staining of wells indicated the presence of neutralizing antibodies. Neutralizing titer was the maximum dilution with a 90% reduction of the CPE, a positive titer was defined as ≥1:10. Positive and negative controls were included in all test runs: Every test included serum control (1:10 dilution), cells control (VeroE6 cells alone) and viral control (three-fold series dilution).

## 3. Results

**Figure 1:**
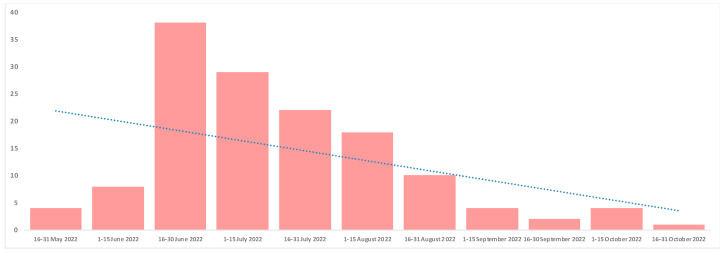
Timeline of mpox cases diagnosed at the Infectious Diseases Unit of San Raffaele Hospital, Milan, Italy. Dotted line: linear trend line.

Overall, 140 individuals were diagnosed with mpox at the Infectious Diseases Unit of San Raffaele Hospital between May and November 2022. The timeline of diagnosed cases is presented in Figure 1.

Individuals were re-assessed every week, depending on clinical judgement, and usually received four to five medical visits and collection of specimens, which could have resulted in occupational transmission. Regarding the clinical characteristics and possible risk factors for mpox transmission to healthcare workers, 108 (77%) had cutaneous lesions and 36 (26%) oral lesions, with 31 (22%) complaining of pharyngitis, and one had ocular lesions. Individuals showed positive mpox PCR on cutaneous swabs and often at the oropharyngeal site. Due to complications, nine (6%) people were hospitalized for a median of six days (interquartile range: 3–7), resulting in prolonged in-hospital stays in dedicated single rooms. Referring to the transmission risk derived from specimen sampling and handling, overall, 1836 samples for mpox testing were collected. In more detail, 590 swabs from cutaneous and mucosal lesions, 405 pharyngeal swabs, 229 urine and 286 seminal fluids samples were collected. Moreover, 326 plasma samples were drawn from individuals with mpox. One individual hospitalized required fibroscopic examination due to laryngeal and pharyngeal involvement, a procedure at high-risk of viral dissemination through aerosol. Overall, 10 individuals required anoscopic examination given the presence of proctitis, which could also facilitate mpox transmission due to contamination with bodily fluids. Following the rapid implementation of a hospital policy aimed at controlling viral transmission, protective equipment was used from the very beginning of the outbreak. All medical personnel and nurses used correctly PPE at every assessment or contact with suspected or infected individuals.

No cases of occupational mpox infection were documented at our center among all healthcare workers working in the Infectious Diseases Unit and among all those providing care for people with suspected or documented mpox infection. Overall, 6/22 medical doctors and 15/29 nurses previously received smallpox vaccination. However, several cases of breakthrough mpox infections among individuals previously vaccinated against smallpox were documented, rendering the expected protection marginal. According to Lombardy regional guidelines for MVA-BN vaccination as pre-exposure prophylaxis, individuals belonging to key populations or handling laboratory samples for virologic analyses were vaccinated and healthcare providers did not receive MVA-BN for an occupational reason. In our center, three medical doctors and one nurse received the MAV-BN vaccine as they fit the non-occupational criteria; no others were vaccinated against mpox. Despite this, no in-hospital infections among medical personnel were to date observed.

One medical doctor accidentally punctured with a scalpel contaminated with blood from a person living with HIV at the time of the mpox diagnosis. Apart from hypertension, the medical doctor was a healthy individual without a history of immune depression, born in 1960 and thus vaccinated in infancy against smallpox. In June 2023, during routine clinical practice, he was visiting an individual with suspected mpox. He was wearing all PPE according to local and international guidelines, including gloves. In order to correctly collect the cutaneous swabs, he scraped aided with a lancet one cutaneous vesicular lesion of the individual. Accidentally, a needlestick injury occurred, with the bloody lancet causing a deep cut on his finger. The individual with suspected mpox received diagnosis confirmation, showing positive mpox PCR on the cutaneous swab (cycle threshold: 20), seminal fluids (cycle threshold: 37) and urine (cycle threshold: 36). Mpox plasma viremia was found to be positive (cycle threshold: 36). Post-exposure vaccination with MVA-BN was not administered to the healthcare worker, as the vaccination campaign was not yet started in Italy at time of exposure. Monitoring of clinical and systemic symptoms, including monitoring fevers twice daily, was indicated. No mpox-related symptoms were observed during 21 days of strict follow-up. Mpox testing was performed after 7 days to rule out transmission, including PCR testing on the oropharyngeal swab, on the lesion and on plasma, which all resulted negative. Six months following exposure, the plaque reduction neutralization test (PNRT) showed the absence of neutralizing mpox antibodies (<1:10), definitively ruling out contagion and suggesting the absence of cross-protective humoral immunity granted by previous smallpox vaccination. 

## 4. Discussion

Overall, we observed no evidence of mpox transmission among healthcare workers working in our Infectious Diseases Unit. This occurred despite the high number of diagnosed mpox cases, the frequent clinical monitoring with re-assessment during medical visits and all the procedures performed, which included bodily fluids sampling. High-risk procedures, such as anoscopic examination and laryngoscopy were also performed in both the in-patient and outpatient setting. We did not observe evidence of infection even following a very high-risk exposure such as a puncture with contaminated blood from an individual with mpox-positive cutaneous lesions and detectable mpox DNA on plasma. Although this exposure was at substantial risk of mpox transmission, both the clinical signs and the mpox testing did not document infection, which was ultimately ruled out by means of serologic testing with undetectable negative neutralizing antibodies. However, cases of occupational transmission following puncture or cutaneous contact have been reported to date [17,18,19,28]. This reinforces the need to strictly adhere to hospital local, national and international protocols, in order to avoid occupational transmission, especially during an epidemic time, where the working load is particularly heavy, and all personnel is paramount for adequate outbreak response. For instance, this was particularly true for the SARS-CoV-2 pandemic [25]. The use of protective equipment, according to guidelines that should be provided very rapidly by the local infection control teams, which help clinicians from the very beginning of new outbreaks, is pivotal. In the current mpox outbreak, very few clinicians had knowledge of mpox, given that this virus was usually diagnosed in endemic countries in Africa. Outbreak and epidemic preparedness and rapid dissemination of guidelines are therefore needed to adhere quickly to the best available evidence on how to reduce transmission risk. Patient management, environmental hygiene protocols, and hospital paths are all crucial to mitigate this risk. In our experience, strict collaboration between motivated clinicians and nurses, members of the infection control team and the diagnostic units significantly contributed to avoiding any risk of occupational transmission in our center. Hospital preparedness for the management of new infectious disease outbreaks, with rapid implementation of policies aimed at controlling infection and strictly adhering to the use of PPE, are paramount to avoid any occupational transmission, ensure adequate response to epidemics and provide safe care to patients.

## Data Availability

The data that support this study are available upon reasonable request to the corresponding authors and are not publicly available due to ethical reasons.
